# Changes in aerobic capacity to prepubertal children in sports 12-month training period

**DOI:** 10.1080/07853890.2025.2514074

**Published:** 2025-06-12

**Authors:** Kęstutis Pužas, Loreta Stasiulė, Arvydas Stasiulis

**Affiliations:** Department of Health Promotion and Rehabilitation, Lithuanian Sports University, Kaunas, Lithuania

**Keywords:** Aerobic, children, football, training, capacity

## Abstract

**Introduction:**

This study was long-term and lasted for one year. The study aims to determine whether there is a change in aerobic capacity indicators for prepubertal children when playing football.

**Methods:**

There were two groups of subjects: children of prepubertal age who trained in football and those who did not attend football. There were 16 participants in the FG (football-trained group) and 15 in the CG (control group) who attended no football lessons. An incremental treadmill test was performed three times with a half-year break to determine the following peak variables: oxygen uptake, stroke volume, cardiac output, and minute ventilation.

**Results:**

After one year of training, the VO2 peak at FG increased from 51.81 ± 6.55 ml/kg/min. to 53.11 ± 5.27 ml/kg/min. (*p* = 0.0412), stroke volume at FG increased from 41.3 ml/min to 46.4 ml/min. (*p* = 0.0012), cardiac output (Q) also increased from 8.15 l/min to 9.44 l/min. At stage II and 10.2 l/min at stage III (*p* = 0.0143) at FG.

**Conclusions:**

After one year of football training at FG-related VO2, RR (-respiratory rate), and Q also shifted, SV (-stroke volume) also increased significantly, while at UG, results were the opposite- only a few parameters, such as HR, increased significantly, while the others did not.

## Introduction

Many authors wrote about maximal oxygen uptake (VO_2_ max) and emphasised that this parameter is the best indicator of an organism’s reaction to physical load [1–3]. Sedentary behaviour is characterised by deficient energy expenditure [4]. However, it is recommended to spend no more than 2 h per day without breaks in front of the screen or a sitting position [5]. These are just some of the examples of scientists emphasizing the benefits and importance of physical activity. Football players must be physically developed because this sport requires a lot of endurance, speed, strength, and coordination skills [6], ass energy in their organisms is mainly produced by aerobic metabolism [7,8]. Several studies have shown that high-intensity training improves soccer players’ fitness levels and skills, such as sprint, strength, and speed endurance [9,10], but most of these studies are done in the short term and with adults. On the other hand, assessing VO_2_ max in children is difficult, sometimes even impossible, due to psychological aspects during the study [11]. In pediatric cardiorespiratory exercise testing, achieving maximal workload is frequently unattainable. Consequently, a significant proportion of tests are prematurely terminated, despite the acquisition of substantial physiological data. Maximum load tests for children depend on each participant’s willingness to continue tests with a relatively high workload when shortness of breath, fatigue and other feelings of stress appear. During the tests, a load is reached, which is usually not reached during natural physical exertion and is not particularly pleasant for most children; therefore, very often, the support of researchers is an encouragement to take the test further [^12^]. It is established that the oxidative capacity of children is higher than that of adults due to faster O2 extraction. Homogenous muscle fiber differentiation with respect to activation and composition in children likely facilitates faster O_2_ extraction. This phenomenon is associated with increased activation and relative dominance of type I motor units. This may contribute to alterations in muscle strength and force balance [13]. This is the main purpose to understand that there are relatively significant differences between the physiology of children and adults.

Genetic factors exert a significant influence on children’s physiological characteristics. However, our understanding of these genetic influences remains limited. Further research, requiring substantial time and financial resources, is necessary to elucidate the complex interplay between genetics and physiological development in children. Authors of this article believes, that participation in a structured football training program will significantly enhance the aerobic capacity of prepubescent children compared to a control group. Given the dynamic nature of sports performance and the evolving understanding of athletic development, it is imperative that coaches possess the knowledge and skills necessary to effectively train young football players [14].

In many countries, children frequently play football [15]. It is essential to conduct appropriate evaluations of peak oxygen uptake, respiratory compensation point, and ventilatory threshold to assess children in soccer and their training progress [16]. VO_2_ is a commonly used parameter to demonstrate training effectiveness [17]. In scientific literature, authors often use ‘VO_2_ peak’ instead of ‘VO_2_ max’ because participants frequently cannot reach maximal aerobic capacity due to factors such as limb fatigue and psychological aspects [18]. However, assessing VO_2_ max in children can be difficult, and sometimes even impossible, due to the psychological aspects of the study [11]. Faster cardiac recovery in prepubertal children during training sessions may be due to more significant reactivation of the parasympathetic autonomic nervous system after exercise [19]. Given the constantly changing landscape of sports achievements and their interpretations, it is crucial to prepare coaches to train children attending football correctly [14]. We hypothesise that long-term football training would positively impact peak variables, including VO2 peak, Q peak, SV peak and other variables.

## Methods

### Participants: inclusion criteria, description of participants

This study is long-term: subjects are studied using the same research methods three times: at the beginning of the study, after half a year and one year after the start. The subjects of this research were selected from the nearest football schools, ‘*Fortūna*’ and ‘*Olimpus*’ after consultation with their coaches to be taken as subjects because they were active. A total of 31 children of prepubertal age participated in this study. There were two groups in this study. One group consisted of children, regularly, at least 90 min a week, training football. It was an investigative football group (-FG) with 16 subjects. During their training, first there was a warm-up, followed by the main part of the training, where the children played in different roles (there are a lot of them in football), after which, at the end of the training, the children were engaged in stretching exercises for several minutes. Subjects in the control group (untrained-UG) did not attend any additional physical activity classes and only attended an ordinary high school. The lessons were held differently in this case since some schoolteachers tell boys to play basketball, girls – volleyball, and so on. Physical education classes are quite different. In the past, the study’s authors did not think it could lead to differences between groups before the study. The children of this group attended only regular physical education classes in high schools. UG consisted of 15 participants. In this place, it is important to understand that the physical activity of children who attended football training (FG) was certainly much higher than that of those who simply attended high schools, without additionally engaging in more intense physical activity.

All the participants of this study were recruited/invited based on the following criteria: (i) they trained at least two sessions per week from 90 to 120 min duration per session for at least one year (allocated FG), (ii) had no cardiac or respiratory diseases (parents or guardians of participants had to submit a doctor’s certificate about the child’s health), (iii)voluntary participation in the study, (iv) was 7–10 years old and (v) attended physical education classes in schools.

Subjects of both sexes were invited to participate in this study, but only a few girls and their parents/guardians responded; therefore, the researchers decided to eliminate them from the study and observe it only for prepubertal boys. Exclusion criteria were as follows: (i) any diseases or illnesses, injuries, or disorders; (ii) any contraindications for physical activity; (iii) no valid doctor’s permission to participate in moderate and high-intensity physical activity classes; and (iv) no or lost voluntary participation.

All participants were divided into two groups: prepubertal children (aged 7–10 years) who regularly participated in football training at least twice per week for one year and prepubertal children who were not trained in physical activity lessons except at school. Descriptive and sociodemographic information about the participants is depicted in Table 1. All parameters were monitored for one year (the study had stage III – at the beginning of the study, after six months, and after 12 months from the start), which means that during this time, participants lived by the usual rhythm of life and practised football (allocated to FG).

**Table 1.** Changes of characteristics, related with aerobic capacity of participants, in one year.

Characteristics		Football group			Control group	
	I phase	II phase	III phase	I phase	II phase	III phase
	mean	mean	mean	mean	mean	mean
Age, yrs	8.2 ± 0.66	8.8 ± 0.64	9.2 ± 0.51	8.18 ± 0.60	8.78 ± 0.53	9.18 ± 0.48
Height, m.	1.32 ± 0.03	1.35 ± 0.03	1.37 ± 0.02	1.33 ± 0.03	1.34 ± 0.03	1.36 ± 0.03
Weight, kg.	32.6 ± 2.5	34.5 ± 1.9	36.2 ± 1.9	33.47 ± 2.35	35.2 ± 2.19	36.3 ± 1.86
BMI (Body Mass Index), kg.	16.4 ± 1.61	16.7 ± 1.32	16.8 ± 1.41	16.7 ± 1.34	17.4 ± 1.65	17.7 ± 1.76
Fat percentage, %	13.8 ± 2.52	13.9 ± 2.47	13.4 ± 2.31	13.9 ± 2.87	14.2 ± 2.51	14.7 ± 2.87
Fat mass, kg	4.17 ± 1.35	4.22 ± 1.32	4.47 ± 1.32	4.28 ± 1.29	4.61 ± 1.33	4.92 ± 1.47
FFM (fat-free mass), kg	25.61 ± 1.73*	25.69 ± 1.78*	26.29 ± 1.84*	25.48 ± 1.94*	25.52 ± 1.94*	25.74 ± 1.95*
TBW (total body water), kg	18.89 ± 1.51	18.93 ± 1.46	19.27 ± 1.39	18.66 ± 1.43	18.57 ± 1.49	18.60 ± 1.51
PAT (High-intensity physical activity time) (h/wk.)	7.13 ± 0.84*	7.41 ± 0.92*	7.43 ± 0.86*	4.39 ± 0.19*	4.53 ± 0.21*	4.18 ± 0.35*

*Note.* Values are presented averaged ± standard deviation.

* Statistically significant differences.

### Measuring before testing

All participants were evaluated at the beginning of all three study stages: age in years and height using a vertical stadiometer (*Leicester HM-250P, Marsden, Rotherham, UK*). The weight of all participants was measured using a body composition analyser (*TBF-300A, Tanita, Tokyo, Japan*). This bioelectric impedance analysis device can measure many body composition parameters [20]. Using this analyser, we measured fat-free mass (FFM), fat mass, fat percentage, and body mass index (BMI). Parents/guardians of participants were asked to fill out the assessment form based on *Tanner* stages [21] to evaluate their maturity level.

### International physical activity Questionnaire

The parents/guardians of the participants were asked to fill out forms about the types of physical activities and their hours.

### Incremental running test to determine submaximal aerobic parameters

**Figure 1.**
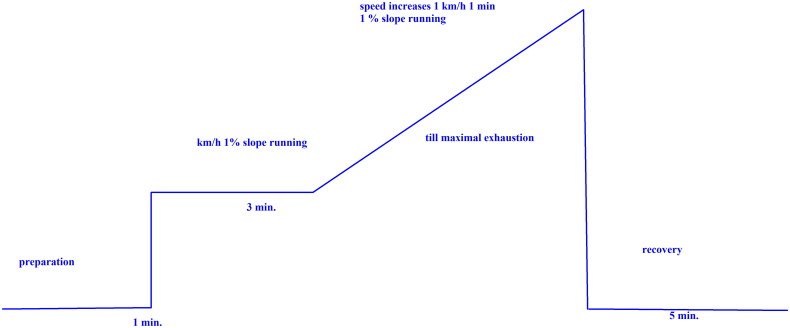
A schematic protocol of the incremental running test.

An incremental running test was performed while running on a treadmill with a portable *Oxycon Mobile* (Germany) O_2_/CO_2_ gas analysis system multifunctional measuring device on the back, the function of which was to record parameters of the heart and respiratory systems during physical exertion [22]. Before the test, the device was calibrated with a gas supply of 180kPA (15.2% O_2_, 5.02% CO_2_ and 79.62% N_2_) to minimise the research error. The subjects had a *Polar Accurax Plus* belt (Polar, Finland) with a heart rate monitor on their chest. The running protocol on a treadmill was modified by Jones and Carter [23]. At the beginning of the test, the treadmill speed was 6 km/h, the slope was 1%, and the subjects had to run at this intensity for 3 min. After 3 min, the treadmill accelerated consistently at 100 m/h – every 6 s. The test was stopped when the subjects felt they could not continue due to fatigue. The test protocol is illustrated in Figure 1. The following submaximal indicators of aerobic capacity assessment and their changes during the entire test were measured: oxygen uptake VO_2_ ml/kg/min and VO_2_ l/min, heart rate (t/min), pulmonary ventilation (VE) (l/min), respiratory rate (RR) (t/min) and volume (l/min), ventilatory thresholds I (VT1) and II (VT2), respiratory exchange ratio (RER). Ventilation thresholds were determined and evaluated as follows: 1. visually increased CO_2_ release compared to O_2_ release; 2. ventilation increases VE/CO_2_ but not VE/VCO_2_ [24]. Cardiac output (Q) and stroke volume (SV) were measured using a *Physioflow device* (PF07 Enduro, Manatec Biomedical, Poissy, France), which can be described as a real-time recording non-invasive device that measures the heart’s work during any exercise and is controlled by a USB cable [25], and places to stick electrodes as recommended by Tonelli et al. [26].

### Statistical analysis

The normality of the data was measured using the *Shapiro-Wilk* test, while the homogeneity of variances was carried out using *Levene’s* test. The confidence level was chosen as 95%. Differences in variables between groups were evaluated separately using an analysis of variance system (ANOVA). We tested all group members three times, so we used a mixed ANOVA test to compare differences by group in each period (1st–3rd phases). The correlation between peak variables and all statistical calculations was performed using IBM SPSS 25.

## Ethics

The study participants and their parents or guardians were fully informed about the essence of the study and its usefulness. In addition, participants and parents/guardians could receive information about the child’s growth and changes in aerobic parameters every 6 months), possible risks (fatigue and exhaustion), the possibility of withdrawing from the study at any time, and the publication and use of the study results based on research ethics and the Declaration of Helsinki. The participants and their parents were informed about the possible inconveniences for the participants: (1) Time spent participating in the study, (2) during exercise and maximal oxygen uptake test on a treadmill: dry mouth and thirst experienced; (3) during the test, feelings of physical difficulty, and fatigue; and (4) heavy sweating during the test.

In Lithuania, scientists are required to obtain permission from the regional Bioethics Committee to conduct biomedical research on humans. The authors of this article are not affiliated with the Bioethics Committee for data clarity and to avoid conflict of interest. The Kaunas Regional Bioethics Committee granted permission for the study (No CE-2011B, date: 07/03/2019). Parents signed a personal consent form. It is essential to mention that according to the laws of the Republic of Lithuania, children under 12 do not have the right to sign documents. For this reason, the children had to give verbal consent and their parents’ consent in writing form with a signature.

## Results

Table 1 shows descriptive and anthropometric characteristics of FG and CG boys. According to the International Physical Activity Questionnaire, FG group members were physically active at approximately 7.13 h/week and grew to 7.43 h/week in one year. While UG, this variable decreased significantly from approximately 4.39 h/week to 4.18 h/week in one year (*p* = 0.046).

The BMI also shifted: UG at the first stage was approx 16.7 kg. In one year, there was a displacement of 17.7 kg. Meanwhile, the FG group showed better results: BMI increased by only 0.4 kg., indicating that subjects were active in sports and their lean body mass increased. The FFM increased from 25.61 to 26.29 kg. At FG, it indicated an increase in lean body mass, which is considered an indirect indicator of aerobic capacity. At UG, it increased from 25.48 to 25.74 kg in one year. Similar results were observed for fat percentage; at UG, it increased from 13.9 to 14.7 per cent. It increased in the first six months only from 13.8% to 13.9%, while in the other half a year, it decreased to 13.4%, which shows the effect of training football in FG.

As shown in Tables 2 and 3, some differences were within the subjects’ groups in a few cardiorespiratory parameters: VO_2_ peak, l/min. at FG increased from 1.518 to 1.647 L/min (*p* = 0.053). At FG-related VO_2_ peak (ml/kg/min) increased from 51.81 to 53.11 ml/kg/min. (*p* = 0.0412), pulmonary ventilation (VE) also increased from 54.32 to 60.21 l/min. (*p* = 0.061), while the UT group showed VO_2_ peak l/min and oxygen uptake from 1.375 l/min. Moreover, 48.00 ml/kg/min increased to 1.41 l/min. And 48.26 ml/kg/min respectively (*p* = 0.154 and *p* = 0.173). There were no significant differences in the HR peak in FG at 6 months and one-year periods (*p* = 0.0562), while in UG, it increased from 196 to 197 (*p* = 0.733). It is important to emphasise that the RER in the FG group did not change significantly at all III stages in one year (*p* = 0.064), and the UG results were also not significant (*p* = 0.0881) (Tables 2 and 3).

**Table 2.** Changes in aerobic capacity parameters at FG.

		I stage	II stage	III stage	*p*
VO_2_ peak; l/min.		1.518 ± 0.195	1.641 ± 0.157	1.647 ± 0.18	0.053*
VO_2_ peak; ml/kg/min.		51.81 ± 6.55	52.14 ± 5.01	53.11 ± 5.27	0.0412
HR peak; bpm		197 ± 5.15	197 ± 4.88	196 ± 4.96	0.0562
RER peak (respiratory exchange ratio)		1.00 ± 0.05	1.00 ± 0.04	1.00 ± 0.05	0.064
VE peak; 1/min		54.32 ± 9.59	59.87 ± 6.28	60.21 ± 5.87	0.061
RR peak; t/min		64.29 ± 8.54	64.03 ± 8.19	62.79 ± 7.42	0.0145

*Note.* Values are presented averaged ± standard deviation.

* Statistically significant differences.

**Table 3.** Changes in aerobic capacity parameters at UG.

	I stage	II stage	III stage	*p*
VO2 peak; l/min.	1.375 ± 0.064	1.394 ± 0.051	1.41 ± 0.094	0.154
VO2 peak related; l/min	48.00 ± 2.43	48.15 ± 2.43	48.26 ± 4.47	0.173
HR peak	196 ± 6.04	196 ± 5.78	197 ± 5.82	0.733
VE peak	50.45 ± 2.50	51.14 ± 2.21	52.48 ± 2.37	0.045

*Note.* Values are presented averaged ± standard deviation.

* Statistically significant differences.

The RR peak in the FG decreased from 64.29 to 64.03 at the I–II stage interval, while at stage III, it decreased to 62.79 (*p* = 0.015), indicating better aerobic fitness. The opposite trend was observed in the UG, where it was 60.23 at the beginning of the study and decreased to 59.31 at stage III (*p* = 0.0537).

**Figure 2.**
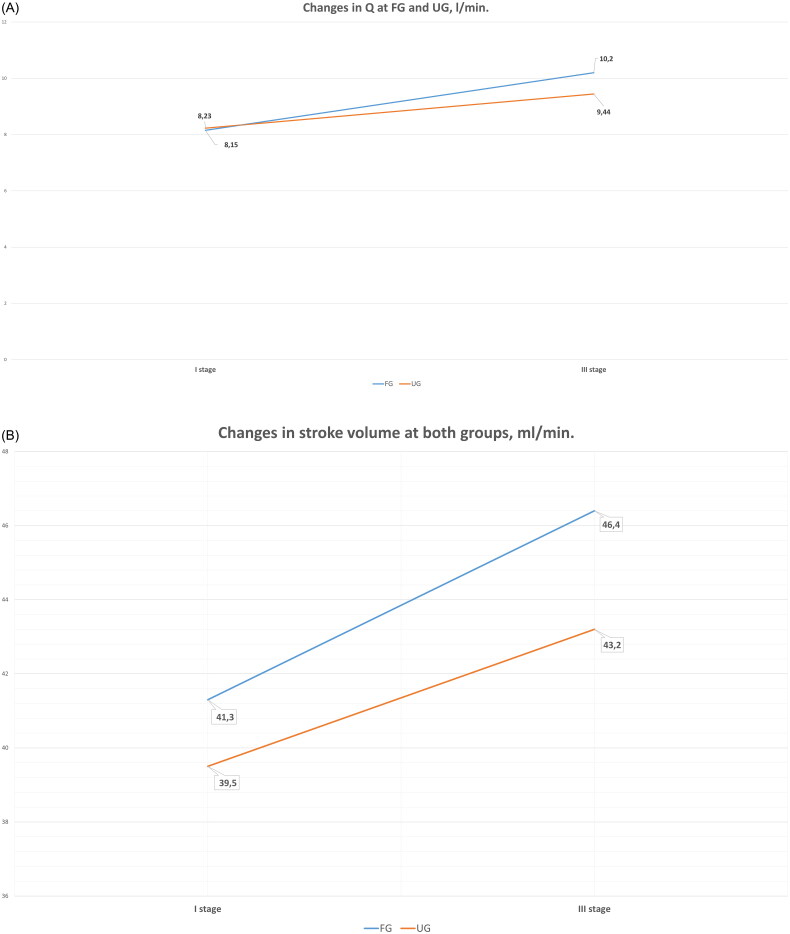
Changes at FG and UG in cardiorespiratory parameters in a year [A – changes in one year at FG and UG in stroke volume; B – changes at FG and UG in cardiac output (Q)]. *Statistically significant differences.

There were also some differences in cardiorespiratory parameters in one year with participation in football training classes (Figure 2A). As we can see in the figure, there was a difference in SV at FG when it increased from 41.3 ml/min at stage I to 46.4 ml/min at stage III (*p* = 0.0012), while at UG, there were no statistical differences in changes at SV (*p* = 0.0592), it increased from 39.5 ml/min at I stage to 42.6 l/min. at stage II stages and to 43.2 ml/min. in the III stage.

There were some differences between groups at Q in FG and UG over the year. As shown in Figure 2B, Q increased from 8.15 l/min to 9.44 l/min at stage II and 10.2 l/min at stage III (*p* = 0.0143) at FG. The year-period results at UG were not significantly different from those at FG: it increased only from 8.23 l/min at the beginning of the study to 9.44 l/min at stage III (*p* = 0.071).

Some differences were at VT1 and VT2 in both the groups. At FG VT1, the results were: at I stage VES1 were 39.3 ± 2.63 L/min and 42.3 ± 4.17 l/min. at III stage, HRS1184 ± 3.16 and 185 ± 6.12 t/min, VO2S11.34 ± 0.029 to 1.54 ± 5.11 L/min at III stage (*p* = 0.044). While at UG, results were not so impressive: VES2 were 37.5 ± 1.78 L/min and increased to 38.4 ± 2.52l/min (*p* = 0.067), HRS2181 ± 4.33 increased to 182 ± 3.76 t/min. (*p* = 0.069) and VO2S2 from 1.32 ± 0.047 at I stage to 133 ± 0.039 at III stage (*p* = 0.059). VES2 increased from 53.16 ± 6.91 to 58.93 ± 6.13 at FG, HRS2 187 ± 5.22 bpm increased to 188 ± 8.42 (*p* = 0.033), VO2S2 increased 1.51 ± 0.063 L/min to 1.63 ± 0.085 (*p* = 0.052). At UG VT2 was different: VES2 increased from 50.27 ± 2.44 to 52.17 ± 3.51 L/min (*p* = 0.0812), HRS2 also shifted from 183 ± 4.66 bpm to 184 ± 6.25 bpm (*p* = 0.096), while VO2S2 increased from 1.36 ± 0.059 L/min to 138 ± 0.073 l/min. at stage III (*p* = 0.071).

One of our objectives was find out or is correlation between aerobic capacity indicators and physical activity hours in a week. As shown in Table 4, we established differences in a few aerobic capacity indicators. VO_2_ peak, SV peak, and VE peak correlated with physical active hours at FG, while at UG, there was a weaker correlation at these indicators.

**Table 4.** Correlation between aerobic capacity indicators and physical activity hours in a week at FG and UG.

Parameter	FG	UG
VO_2_ peak, l/min	0.619*	0.574*
VO_2_ peak related ml/min/kg	0.0641	0.06
HR	−0.28	−0.173*
SV	0.504*	0.511*
Q peak	0.472	0.475
VE	0.612*	0.615
RR	0.062	0.247

*Note*. Values are presented averaged ± standard deviation.

* Statistically significant differences.

## Discussion

Our investigation is partially successful. Summing up, we can note that, as we expected, there are some positive changes in the indicators of aerobic capacity in children of prepubertal age who are engaged in football. Meanwhile, the results of untrained children were surprising. They were not as positive as they were with kids involved in sports. Our objectives were to assess the changes in aerobic capacity indicators, compare the changes in both groups and find out if there is a correlation between aerobic capacity indicators and physical activity hours in a week in both groups. In this part of the article, we will look at everything in this way.

Scientists in many countries recognize body mass index (BMI) as one of the best indicators of body condition. We found that BMI increased at FG only a few points, from 16.4 to 16.8 kg at the end of measurements; it is not surprising that fat percentage decreased slowly from 13.8% to 13.4% due to football training. Meanwhile, BMI at UG increased from 16.7 to 17.4 and 17.7 kg, respectively, meaning that a sedentary lifestyle influences BMI even in prepubertal children. Positive changes at FG were also observed in FFM, which indicates lean mass in kilograms; it increased significantly from 25.61 to 26.29 kg in one year of training football. Opposite results were obtained by Schomoller et al. (2021), where FFM decreased after a training program. Some researchers suggest that FFM is associated with fundamental motor skills [27]. This finding confirms the need for long-term studies and research.

Maximal and submaximal lung ventilation tended to increase in this study’s case of aerobic exercise. Our study shows positive and statistically significant changes in FG football training over a year, which means that a more extended investigation would show a better result for this parameter by football training. RR at FG also decreased significantly and indirectly shows the relationship between physical activity and aerobic capacity indicators by the improved blood flow from the heart to the lungs.

The related VO_2_ peak at FG increased significantly from 51.81 ml/min at Stage I to 52.14 ml/min at Stage II and 53.11 ml/min. At Stage III, relative oxygen consumption is essential to the efficiency of the body’s vital functions during exercise [28]. Therefore, it can be assumed that the relative oxygen consumption in the FG is higher because of the relatively intensive football training. Thus, it can be assumed that FG boys are better equipped and aerobically adapted to greater physical exertion and daily activity than UG boys. At the VO_2_ peak l/min, there were no significant differences between the groups. Some authors have emphasized that the hypothalamus controls the secretion of hormones responsible for glucose metabolism and energy production. Hormones released during stress directly affect aerobic capacity by neural and hormonal factors such as catecholamines, cortisol, glucagon, growth hormone, and insulin [29]. Therefore, most of the research around the world on this topic is performed with adults, and it is tough to argue that precisely these mechanisms lead to better adaptation to exercise at FG. RR is another critical focus in assessing subjects’ health and their indicators during physical activity [30]. The RR at FG was significantly decreased in this study from 64.29 times per minute to 62.79 times per minute. These results suggest an indirect relationship between VO_2_ peak and hypervolemia, which increases systolic blood volume and heat tolerance [31]. On the other hand, there are considerations in another scientific field that VO_2_ peak stabilises over time and changes only because of the cost-effectiveness of work or physical exercise, so it is challenging to make precisely such assumptions. However, differences in VT1 and VT2 at FG suggest that an increase in angiogenesis may determine the cardiovascular system’s better adaptation. Angiogenesis, resulting from physical activity, is regulated by a balance between angiogenic and angiostatic factors. Soccer training in prepubertal children increases capillary density in muscle fibres, indirectly improving aerobic capacity [32].

The HR peak had rather unusual results in the UG. As mentioned in previous chapters, it decreased at the UG from 196 times per minute to 197 times at Stage III. This means that HR is indirectly related to changes in aerobic capacity since the heart has to contract more times to pump the same (relative) amount of blood. This may be related to the sedentary lifestyle in the UG [33]. While RER in both groups did not change significantly, its high values indicate how successfully carbohydrates are used during exercise [34]. Therefore, it could be considered a successful energy use during exercise at FG. On the other hand, RR also decreased at FG; this parameter does not directly show the condition of a person’s health [35]. Similar results were reported in 2011, where the authors emphasised that better HR adaptation results are obtained from training in judo or other endurance sports [36]. These results indicate that more blood is pumped during one cardiac cycle in the body of FG group members, which indicates an improved aerobic adaptation to physical exertion. SV is considered to determine tissue perfusion and systemic oxygenation; therefore, it is not surprising that it is considered one of the best standards for assessing the state of the body. SV at FG increased from 41.3 to 46.4 ml/min, showing the possibility of a more muscular heart contraction. On the other hand, SV is one of the most critical indicators of aerobic capacity in all sports. Therefore, the study time and sample size must be significant to draw such conclusions. This causes financial and human resources difficulties, depending on the country in which it is conducted [3]. While Q at FG increased from 8.15 to 10.2 l/min, these results suggest that it depends on β-1 adrenergic receptors at the heart, which cause positive inotropic (increasing contractility) and negative chronotropic (reducing HR) effects. For this reason, the minute volume of the heart and oxygen demand in the myocardium increase. It constricts arterioles in the skin, mucous membranes, and internal organs, acts through α-1 adrenoreceptors and dilates blood vessels in the muscles and liver, which can be considered an adaptation of aerobic capacity in prepubertal children dependent on training [37].

Many scientific studies consider a sedentary lifestyle as a factor that causes serious problems for human health; therefore, one of the tasks of this study was to evaluate the correlation between physically active hours and some of the aerobic parameters, such as VO_2_ peak-related VO_2_ peak, HR, SV, and so on. One of our objectives was to evaluate possible correlations between hours spent physically active in both groups of children and specific indicators of aerobic capacity. This study found that there may be a positive correlation between physically active hours spent per week and VO_2_ peak, SV and VE. However, it is not very easy to explain the putative causal mechanisms for this. Some authors suggest that one possible proposed mechanism is that the increase in cardiac contractility and the associated increase in aerobic capacity during physical activity are related to insulin and insulin-like growth factor 1 (*IGF1*) [38].

The authors of this work agree that the sample of subjects was too small. Perhaps it is also worth discussing the reliability of the results. Although statistics have found a connection, and it can be argued that the reliability of the data is not affected by a small sample, the scientific world has one feature - scientists always ask why and are always looking for causality. On the other hand, when studying children of this age, ensuring the duration of their physical activity is essential. That’s a very big limit that we’ve faced. Perhaps that limit would be decisive if the investigation sample were more significant. For this reason, as many subjects as possible should be involved in future similar studies.

## Conclusions

Over one year of training, VO_2_ increased in football-trained children. The RR, SV and Q indicators of aerobic capacity also increased. Meanwhile, the results of UG were somewhat different; during one year, many indicators did not change significantly for these prepubertal children, except for HR, which even increased. Given the relatively small sample size and the year-long duration of the study, further research with larger cohorts is warranted. Nevertheless, the authors contend that these limitations do not diminish the academic significance and innovative nature of the present investigation/article.

## Data Availability

By this statement, we agree to make the data and materials supporting the results or analyses presented in our paper available only upon individual request, as the data relates to susceptible information about young children.
